# The Cause of Medicine

**Published:** 1921-04-09

**Authors:** 


The Workers' Newspaper of Administrative Medicine and Institutional Life, Administration, National Insurance and Health.

No. 1818, Vol. LXX. SATURDAY, APRIL 9, 1921. PRICE TWOPENCE

### THE CAUSE OF MEDICINE.

In connection with last week's list of new ministerial appointments, it is hard to let pass unchallenged the view expressed in certain quarters that Dr. Addison's departure from the Ministry of Health is a matter for general satisfaction. Few departments have been faced with more multitudinous or more difficult post-war problems, and Dr. Addison's highly technical work is deserving of more graceful appreciation than some of our contemporaries admit. The outcry concerning expenditure and bureaucratic interference which has accompanied every action of the Department during his more recent period of office appears to us to arise from an almost deliberate misunderstanding of facts (see p. 31).

Never has there been a time more difficult for the inauguration of administrative health measures, and we trust that the future will give his successor, Sir Alfred Mond, more unhampered opportunity. Among so many health matters now under Departmental consideration, it is difficult for general interest to select one more than another. But there is no doubt that the Committee engaged in formulating plans by which London can become a genuine centre of post-graduate medicine is dealing with one of the vital points in reconstruction of the English health service. Whatever the ultimate method, the question of post-graduate instruction must be solved. Great direct and indirect benefits to the public are foreshadowed in any successful scheme, for not only is the family doctor concerned, but the medical officers of the Navy, Army and Air Force, those of the Colonial services, and a host of others who need to be introduced periodically to new methods and technique developed since they were last in touch with the training centres. Emphatically the ideal of bringing London into this position in the medical world is not simply that of meeting the needs of a few young men pursuing a specialised medical education. The difficulties ahead are great, but nevertheless we sincerely hope that in some way it will be possible, gradually if not immediately, to devote a hospital of first-class standing to this one object. Such a centre would be as invaluable to the cause of medicine as to the people in every part of the Empire.

